# VelC Positively Controls Sexual Development in *Aspergillus nidulans*

**DOI:** 10.1371/journal.pone.0089883

**Published:** 2014-02-28

**Authors:** Hee-Soo Park, Tae-Young Nam, Kap-Hoon Han, Sun Chang Kim, Jae-Hyuk Yu

**Affiliations:** 1 Department of Bacteriology, University of Wisconsin, Madison, Wisconsin, United States of America; 2 Department of Genetics, University of Wisconsin, Madison, Wisconsin, United States of America; 3 Department of Pharmaceutical Engineering, Woosuk University, Wanju, Republic of Korea; 4 Department of Biological Sciences, Korea Advanced Institute of Science and Technology, Dae-Jon, Republic of Korea; Soonchunhyang University, Republic of Korea

## Abstract

Fungal development and secondary metabolism is intimately associated via activities of the fungi-specific *velvet* family proteins including VeA, VosA, VelB and VelC. Among these, VelC has not been characterized in *Aspergillus nidulans*. In this study, we characterize the role of VelC in asexual and sexual development in *A. nidulans*. The *velC* mRNA specifically accumulates during the early phase of sexual development. The deletion of *velC* leads to increased number of conidia and reduced production of sexual fruiting bodies (cleistothecia). In the *velC* deletion mutant, mRNA levels of the *brlA*, *abaA*, *wetA* and *vosA* genes that control sequential activation of asexual sporulation increase. Overexpression of *velC* causes increased formation of cleistothecia. These results suggest that VelC functions as a positive regulator of sexual development. VelC is one of the five proteins that physically interact with VosA in yeast two-hybrid and GST pull down analyses. The Δ*velC* Δ*vosA* double mutant produced fewer cleistothecia and behaved similar to the Δ*vosA* mutant, suggesting that VosA is epistatic to VelC in sexual development, and that VelC might mediate control of sex through interacting with VosA at specific life stages for sexual fruiting.

## Introduction

The genus *Aspergillus* is found ubiquitously in our environment and some species are of tremendous importance to humankind as serious human and plant pathogens and as agricultural aids [1]. All *Aspergillus* species commonly reproduce by forming asexual spores called conidia, which are the primary means of infecting host organisms. Conidia also can contain potent allergens and toxic secondary metabolites called mycotoxins [2]. Previous studies proposed that production of some mycotoxins including the most potent natural carcinogens, aflatoxins, is tightly correlated with asexual development (conidiation) [2]–[4]. *Aspergillus nidulans* has served as an excellent model system for studying the mechanisms of asexual development and secondary metabolism [5]–[7].

The *velvet* family proteins, including VosA, VeA, VelB and VelC, have been identified as key regulators that bridge spore formation and mycotoxin production in *Aspergillus* [8]–[10]. In addition, some *velvet* proteins form cell-type specific complexes that play differential roles in controlling fungal biology in *A. nidulans* [9], [11]. In vegetative cells, the VelB-VeA hetero-complex is required for sexual development and production of the mycotoxin sterigmatocystin (ST) by interacting with LaeA [9], [12], [13]. During conidiation, the VelB-VosA hetero-complex plays a key role in maturation, dormancy and germination of spores [12]. The *velvet* homologues are found in most filamentous fungi and have been reported to regulate development and mycotoxin production in other *Aspergilli* [8], [10], [14]. In *Aspergillus fumigatus*, VeA represses conidiation, and VelB and VosA control conidial trehalose amount and conidial germination [15], [16]. In *Aspergillus flavus*, VeA and VelB are involved in the regulation of conidial production and sclerotia formation [17]–[19]. VeA also regulates the biosynthesis of secondary metabolites, including aflatoxin in *A. flavus* [20] and *A. parasiticus* [21], gliotoxin in *A. fumigatus* [16], and penicillin in *A. oryzae* [22].

Recent studies have revealed that the *velvet* proteins control various biological processes by acting as transcription factors [23], [24]. The conserved *velvet* domain forms a novel DNA-binding motif structurally similar to the Rel homology domain (RHD) of the mammalian transcription factor NF-kB. The *velvet* domain in VosA or the VosA-VelB heterodimer recognizes the specific sequences present in the promoters of developmental regulatory genes and controls their expression [24]. In the human pathogen *Histoplasma capsulatum*, the VosA and VelB orthologues Ryp2 and Ryp3 directly bind to a *cis*-acting element and activate expression of temperature-responsive target genes [23], [25]. These results indicate that the *velvet* proteins are fungal specific transcription factors with DNA-binding activity.

While we now have a better understanding on the roles of three *velvet* regulators VeA, VelB and VosA, the function of VelC remains unanswered in *A. nidulans*. In this study, we characterized the roles of VelC in regulating development in *A. nidulans*. While the deletion of *velC* results in reduced cleistothecia production, the overexpression (OE) of *velC* causes enhanced formation of cleistothecia indicating that VelC is a positive regulator of sexual development. We further show that VosA is epistatic to VelC in most biological processes and that VelC physically interacts with VosA in yeast and *in vitro*. Finally, a genetic model depicting the differential roles of the *velvet* regulators in controlling development in *A. nidulans* is presented.

## Materials and Methods

### Strains and culture conditions

*A. nidulans* strains used in this study are listed in Table 1. Individual strains were grown on solid or liquid minimal medium with appropriate supplements (simplified as MM) as described previously [26]–[28] and incubated at 37°C. Medium enhancing sexual development (pH 6.5; 20 g/l glucose, 1,5 g/l glycine, 0.52 g/l MgSO_4_ 7H_2_O, 0.52 g/liter KCl, 1.52 g/l KH_2_PO_4_, and 1 ml/l of 1000 x trace element solution composed of 22 g/l ZnSO_4_7H_2_O, 11 g/l H_3_BO_3_, 5 g/l MnCl_2_4H_2_O, 5 g/l FeSO_4_7H_2_O, 1.6 g/l CoCl_2_5H_2_O,1.6 g/l CuSO_4_5H_2_O, 1.1 g/l (NH_4_)_6_Mo_7_O_24_4H_2_O, 50 g/l Na_2_EDTA; simplified as SM) was used for cleistothecia development test. To determine the numbers of conidia and cleistothecia, wild-type (WT), relevant mutants, and complemented strains were point inoculated and grown on solid MM or SM at 37°C for 4 or 7 days.

**Table 1:** *Aspergillus* strains used in this study.

Strain name	Relevant genotype	References
FGSC4	*A. nidulans* wild type, *veA^+^*	FGSC^a^
FGSC26	*biA1*; *veA1*	FGSC^a^
FGSC33	*biA1*; *pyroA4*; *veA1*	FGSC^a^
RJMP1.59	*pyrG89*;*pyroA4*;*veA* ^+^	[52]
TNJ36	*pyrG89* Afu*pyrG* ^ +^; *pyroA4*;*veA* ^+^	[38]
THS8.1	*biA1*; *pyroA*::*alcA*(p)::*velC*::FLAG:: *pyroA* b; *veA1*	This Study
THS11.1	*pyrG89*; *pyroA4*; Δ*velC*::Afu*pyrG* ^ +^; *veA* ^+^	This Study
THS15.1	*pyrG89*; *pyroA4*; Δ*vosA*::Afu*pyrG* ^ +^; *veA* ^+^	This Study
THS23.1	*pyrG89*; *pyroA*::*nii*(p)::*velC*:: FLAG::*pyroA* b; Δ*velC*::Afu*pyrG* ^ +^;*veA* ^+^	This Study
THS25.1	*pyrG89*; *pyroA*::*velC*(p)::*velC*::FLAG_3x_::*pyroA* b; Δ*velC*::*AfupyrG* ^ +^; *veA* ^+^	This Study
THS26.1	*pyrG89*; *pyroA4*; Δ*vosA*::*pyroA^+^*, Δ*velC*::Afu*pyrG* ^ +^; *veA* ^+^	This Study

a Fungal Genetic Stock Center

b The 3/4 *pyroA* marker causes the targeted integration at the *pyroA* locus.

To examine the effects of OE of *velC* by an ectopic copy of *velC* under the *alcA* promoter [29], [30], all strains were inoculated on solid MM with 1% glucose (MMG, non-inducing) or MM with 100 mM threonine as a sole carbon source (MMT to induce OE of *velC*) at 37°C for 7 days. Effects of OE of the *velC* gene under the *niiA* [31] promoter in were examined by growing the strains in both MM with 0.2% (w/v) ammonium tartrate (MM + AT, non-inducing) and MMG (inducing, containing 0.6% (w/v) sodium nitrate).

For Northern blot analyses, samples were collected as described [32]. Briefly, for vegetative growth, conidia (5×10^5^ conidia/ml) of WT and mutant strains were inoculated in 100 ml liquid MM in 500 ml flasks and incubated at 37°C. Samples of liquid submerged culture were collected at designated time points, squeeze-dried and stored at −80°C. For sexual and asexual developmental induction, 18 h vegetatively grown mycelia were filtered, washed and transferred to solid MM and the plates were air exposed for asexual developmental induction or tightly sealed and blocked from light for sexual developmental induction [32].

*Saccharomyces cerevisiae* L40 strain was grown on the synthetic dropout (SD) minimal medium with various supplements (10 ml of 100X nutrient solution containing 10 g/l leucine, 2 g/l tryptophan or 2 g/l histidine) [33]. *Escherichia coli* strains, DH5α and BL21 (DE3), were grown in Luria–Bertani medium with ampicillin (50 mg/ml) for plasmid amplification.

### Generation of the velC mutants

The oligonucleotides used in this study are listed in Table 2. For the deletion of *velC*, Double-Joint PCR (DJ-PCR) method was used [34]. Both 5′ and 3′ flanking regions of *velC* were amplified using the primer pairs OMN137;OMN141 and OMN138;OMN142 and *A. nidulans* FGSC4 genomic DNA as a template. The *A. fumigatus pyrG*+ marker was PCR-amplified from *A. fumigatus* AF293 genomic DNA with the primer pair OJH84;OJH85. The final DJ-PCR *velC* deletion construct was amplified with OMN139;OMN140. The deletion cassette was introduced into RJMP1.59 (Table 1) protoplasts generated by the Vinoflow FCE lysing enzyme (Novozymes) [35]. To generate the double deletion mutants, 5′ and 3′ flanking regions of *vosA* were amplified using OMN54;OHS184 and OMN55;OHS185. The *pyroA*+ marker was amplified from FGSC4 genomic DNA with the primer pair ONK395;ONK396. After the fusion by DJ-PCR, *vosA* deletion construct was amplified using OMN58;OMN59 and introduced into THS11.1 (Table 1). Multiple (at least three) deletion mutants were isolated and confirmed by PCR followed by restriction enzyme digestion in each case.

**Table 2:** Oligonucleotides used in this study.

Name	Sequence (5′ → 3′)^a^	Purpose
OJA142	CTGGCAGGTGAACAAGTC	5′ *brlA* probe
OJA143	AGAAGTTAACACCGTAGA	3′ *brlA* probe
OJA150	CAGTACGTCAATATGGAC	5′ *wetA* probe
OJA151	GTGAAGTTGACAAACGAC	3′ *wetA* probe
OJA154	AGCTCTTCAGAATACGTC	5′ *abaA* probe
OJA155	GTTGTGAGATGCCTCCAT	3′ *abaA* probe
OMN66	TTTCCAGATCCTTCGCAG	5′ *vosA* probe
OMN63	ATAGAAACAGCCACCCAG	3′ *vosA* probe
OHS127	AATTGAATTCGATGACCACCCACGTGGGCC	5′ *velC* probe
OHS128	AATTAAGCTTCTATTCAACTCGAGCCCTCGAAGAT	3′ *velC* probe
OJH84	GCTGAAGTCATGATACAGGCCAAA	5′ *AfupyrG* marker
OJH85	ATCGTCGGGAGGTATTGTCGTCAC	3′ *AfupyrG* marker
ONK395	ATCTCATGGGTGCTGTGCGAAAGG	5′ *pyroA* marker
ONK396	TTGCATCGCATAGCATTGCATTGC	3′ *pyroA* marker
OMN137	CCGCAAGATCTACAGAGCACAG	5′ flanking region of *velC*
OMN138	GTGCCATGGACATCAGAGTATC	3′ flanking region of *velC*
OMN139	TGACAAACTGGCGACTGTTCTC	5′ nested of *velC*
OMN140	TCAAGGCCTACGAGGTCATTAC	3′ nested of *velC*
OMN141	*GGTGAAGAGCATTGTTTGAGGCA* GCGGTCGTTGGGTGCTTATAAT	5′ *velC* with *AfupyrG* tail
OMN142	*AGTGCCTCCTCTCAGACAGAATA* ATGTTTTGAGGGACTCCAACTC	3′ *velC* with *AfupyrG* tail
OMN54	TTTTTGCCGCTGCTGGAGTTAG	5′ flanking region of *vosA*
OMN55	AAGAGGGCTTTGTGGGGTTTTC	3′ flanking region of *vosA*
OMN58	GCTATAACAAAGAGAGAGAGGG	5′ nested of *vosA*
OMN59	TTCGAAAAATATGCCGGGGCTG	3′ nested of *vosA*
OHS184	*ACTTCTGCAGTCGGAATTGGCCTG* GAGCACTATGAGAGACGACTG	5′ *vosA* with *pyroA* tail
OHS185	*TGGTGAGAACACATGCACAACTTG* GGATTCTCGTTTGTGGAACAC	3′ *vosA* with *pyroA* tail
OHS166	*GGTGAAGAGCATTGTTTGAGGCA* GAGCACTATGAGAGACGACTG	5′ *vosA* with *AfupyrG* tail
OHS167	*AGTGCCTCCTCTCAGACAGAATA* GGATTCTCGTTTGTGGAACAC	3′ *vosA* with *AfupyrG* tail
OMN304	CGG**GAATTC**ATGACCACCCACGTGGGCCCTC	5′ *velC* with *Eco*RI
OHS178	AATT**GAATTC**GATACCGCAATCCTTAGGTGATCCG	5′ *velC* with *Eco*RI
OHS179	AATT**AAGCTT**TTCAACTCGAGCCCTCGAAGATAC	3′ *velC* with *Hin*dIII
ONK114	TCTATTCGATGATGAAGATACC	5′ pADGal4
ONK115	TCATAGATCTCTGCAGTAATAC	3′ pADGal4
OMN329	CGG**GAATTC**ATGAGTGCGGCGAACTATCCAG	5′ *vosA* with *Eco*RI
OMN330	ACGC**GTCGAC**CAAGCCAGTCAATTAGGTGCATAG	3′ *vosA* with *Sal*I
OMN304	CGG**GAATTC**ATGACCACCCACGTGGGCCCTC	5′ *velC* with *Eco*RI
OMN305	ATAT**GCGGCCGCC**TATTCAACTCGAGCCCTCGAAGA	3′ *velC* with *Not*I
OMN306	CGG**GAATTC**ATGAAGGCCTTCAGCTACGAGACG	5′ *voiA* with *Eco*RI
OMN307	ATAT**GCGGCCGCC**TCACTTCCAGCTCATCTCTCCAAG	3′ *voiA* with *Not*I
OMN308	CGG**GAATTC**ATGCTCACGACCAGGCGAAACCAT	5′ *voiB* with *Eco*RI
OMN310	ATAT**GCGGCCGCC**TTACGCGGCGAGTGAACGCTTGGT	3′ *voiB* with *Not*I
OMN313	CGG**GAATTC**ATGTCTGGCCCCTACGATCACAAC	5′ *voiD* with *Eco*RI
OMN314	ATAT**GCGGCCGCC**TCATTTCTTGAAGAAGCTGCCGAG	3′ *voiD* with *Not*I
OMN315	CG**GGATCC**ATGGCATCGGCGGTTTTCTTCCTA	5′ *voiC* with *Bam*HI
OMN316	ATAT**GCGGCCGCC**TCATTGTACATCCGGCATTCGGAC	3′ *voiC* with *Not*I

a Tail sequence is in italic, Restriction enzyme site is in bold.

To complement Δ*velC*, the WT *velC* locus including its predicted promoter and coding region was amplified with the primer pair OHS178;OHS179, digested with *Eco*RI and *Hin*dIII and cloned into pHS13 [12], which contains ¾*pyroA* [36], a 3xFLAG tag and the *trpC* terminator [37]. The resulting plasmid pHSN32 was then introduced into the recipient Δ*velC* strain THS11.1, in which preferentially a single copy *velC^+^* gets inserted into the *pyroA* locus complementing the *pyroA4* allele, and gives rise to THS25.1.

To generate the *alcA*(p)::*velC* or *niiA*(p)::*velC* fusion construct, the *velC* ORF derived from WT genomic DNA was amplified using the primer pair OMN304;OHS179. The PCR product was then double digested with *Eco*RI and *Hin*dIII and cloned into pHS3, which has the *alcA* promoter and the *trpC* terminator [38], or into pHS11 that contains the *niiA* promoter and the *trpC* terminator. The resulting plasmids pHSN7 (*alcA*(p)::*velC*) and pHSN12 (*niiA*(p)::*velC*) were then introduced into FGSC33 and THS11.1, respectively. The *velC* overexpression (OE*velC*) strains among the transformants were screened by Northern blot analysis using a *velC* ORF probe followed by genomic DNA PCR confirmation for the presence of OE alleles.

### Nucleic acid isolation and manipulation

To isolate genomic DNA, about 10^6^ conidia of WT and mutant strains were inoculated in 2 ml liquid MMG + 0.5% yeast extract, and stationary cultured at 37°C for 24 h. The mycelial mat was collected, squeeze-dried, and genomic DNA was isolated as described [34], [39]. Total RNA isolation and Northern blot analyses were carried out as previously described [34], [40], [41]. To examine the positions of introns, *velC* cDNA was synthesized from total RNA and sequencing analyses of *velC* were carried out. The DNA probes were prepared by PCR-amplification of the coding regions of individual genes with appropriate oligonucleotide pairs using FGSC4 genomic DNA as a template (Table 2).

### Yeast two-hybrid assay

The *LexA* based yeast two-hybrid system permitting to detect the *LacZ* reporter gene expression on the screening plates with X-gal was used. The cDNA of *vosA* coding region was cloned between *Eco*RI and *Sal*I of pTLexA [42] (kindly provided by Suhn-Kee Chae at Paichai University, Daejeon, Korea), which contains the yeast *TRP1* selection marker and Zeocin resistance gene. The resulting plasmid pNI39 (bait vector) was introduced into the *S. cerevisiae* reporter strain L40 (Invitrogen) using lithium acetate-polyethylene glycol-mediated yeast transformation [43]. Then, the *A. nidulans* cDNA library in pAD-GAL4-2.1 (prey vector; provided by K.-Y. Jahng, Chunbuk University, Jeonju, Korea) was screened. The transformants were directly selected on SD plates (-his, -trp, -ura, -leu) with 1 mM 3-amino-1, 2, 4-triazole (3-AT). The big colonies were further transferred to SD plates (-trp, -ura, -leu) with 80 mg/L X-gal, and the colonies showing intense blue color after incubation were picked. Yeast genomic DNA was isolated from these candidates, and used for transformation of *E. coli* to recover the prey plasmids by selecting on LB media with ampicillin. Each recovered prey and the pNI39 bait plasmid were further co-introduced back into L40 to confirm they still expressed reporter genes. By direct sequencing of the insert ends of the plasmids of interest with the primer set ONK114 and ONK115 followed by the genome search (the Broad Institute), the potential VosA interacting proteins were identified.

### GST pull down

The *vosA* cDNA ORF was amplified by the primer pair OMN329:OMN330 using the *A. nidulans* cDNA library. The resulting amplicon was purified and digested with *Eco*RI and *Sal*I. The digested *vosA* amplicon was cloned into pGEX 5X-1 (GE healthcare). The resulting plasmid pNI47 was introduced into *E. coli* BL21(DE3) to express GST-VosA. *E. coli* was grown up to O.D. A600  = 0.5∼0.6 at 37°C, 250 rpm, and 0.1 mM IPTG was added for inducing fusion protein expression. The GST fusion protein expression and purification was carried out following the manufacturer's instruction. For concentration and buffer exchange, Amicon Ultra Centrifilter Unit (Millipore) was used. BCA Protein Assay Kit (Pierce) was used to estimate protein concentration.

For expressing *vosA* interacting (Voi) proteins *in vitro*, cDNAs of the coding regions of *velC*, *voiA*, *voiB*, *voiC* or *voiD* were amplified via RT-PCR and cloned between the *Eco*RI and *Not*I (for *velC*, *voiA*, *voiB* and *voiD*) or *Bam*HI and *Not*I (*voiC*) sites in pcDNA3 (Invitrogen) resulting in pNI42, 43, 44, 45 and 46, respectively. pNI42, 43, 44, 45 or 46 was translated *in vitro* by TNT T7 quick coupled transcription/translation system (Promega). Briefly, plasmid was incubated with 20 µCi of [^35^S]-methionine (PE) in TNT mastermix for 90 min at 30°C. Equal amounts of *in vitro* translated proteins were added to Glutathione bead-GST-VosA or Glutathione bead-GST (control) suspensions. The mixture was incubated on a mixer at 4°C overnight. After washing three times with lysis buffer, the samples were mixed with Laemmli sample buffer (Bio-Rad) and loaded to SDS-PAGE gel. The gel was dried down under vacuum to 3 layers Whatman 3MM filter paper. Autoradiography was performed at −80°C with Kodak XAR film

### Microscopy

The colony photographs were taken by using a Sony digital camera (DSC-F828). Photomicrographs were taken using a Zeiss M^2^ BIO microscope equipped with AxioCam and AxioVision digital imaging software (Zeiss).

### Statistical analysis

Statistical differences between WT and mutant strains were evaluated with student's unpaired *t*-test (2-tailed). Mean ±SD are shown. P values <0.05 were considered significant.

## Results

### Summary of VelC

**Figure 1:**
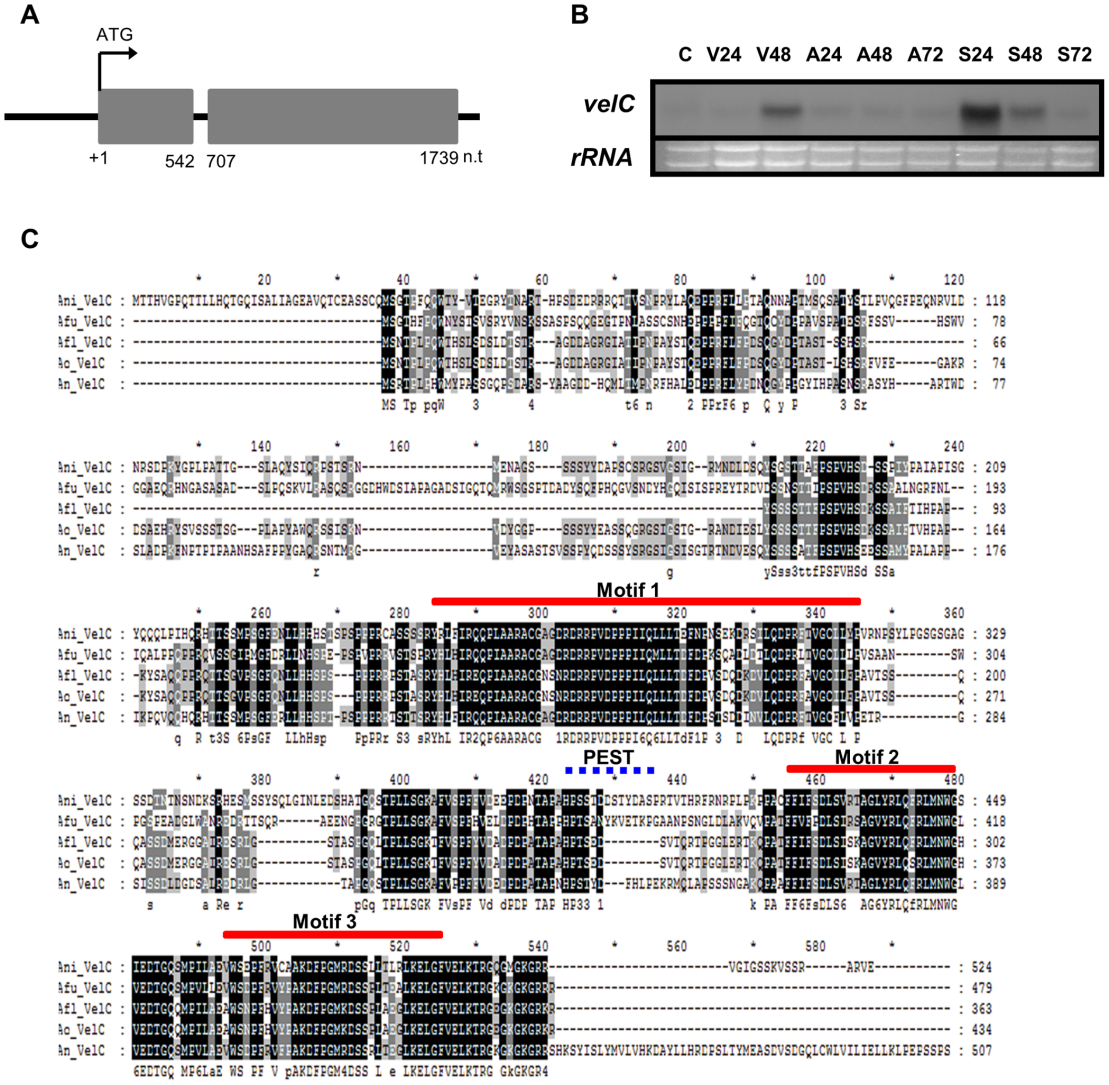
Summary of *velC*. (A) Schematic presentation of the *velC* ORF (shaded box) with a intron (shown by discontinuity in the box). Gene structures were verified by sequence analyses of cDNA of *velC*. Start codon is assigned as 1 (Top). Domain architecture of the VelC in *A. nidulans* (Bottom). (B) Northern blot showing level of *velC* mRNA during the lifecycle of *A. nidulans* WT (FGSC4). Conidia (asexual spores) were indicated as C. The time (hours) of incubation in liquid submerged culture and post asexual (A) or sexual (S) developmental induction is shown. Equal loading of total RNA was confirmed by ethidium bromide staining of rRNA. (C) Alignment of the VelC protein in *Aspergillus* spp., *A. nidulans* (Ani; AN2059), *A. fumigatus* (Afu; Afu4g09770), *A. flavus* (Afl; AFL2G_01807), *A. oryzae* (Ao; AO090003001252), and *A. niger* (An; An04g07320). The conserved motifs are marked by red lines. The PEST domain in *A. nidulans* VelC was marked by a dotted line. ClustalW (http://align.genome.jp/) and BoxShade 3.21 (http://www.ch.embnet.org/software/BOX_form.html) were used for the alignment.

The *A. nidulans velC* gene (EF540816) is composed of a 1,739-bp ORF with one 164-bp intron and predicted to encode a 524-amino-acid polypeptide with a calculated mass of 57.3 kDa (Fig. 1A). To begin to characterize the *velC* gene, we checked levels of *velC* mRNA during the lifecycle by Northern blot. As shown in Fig. 1B, the *velC* transcript is detectable at 48 h of vegetative growth and early phases (24∼48 h) of sexual development, but not during asexual development, suggesting that it may play a certain role in sexual development. The predicted VelC protein (ABQ17968) contains one *velvet* domain in the C-terminal region (252^nd^∼501^st^ aa), which is highly conserved in *Aspergillus* spp (Fig. 1C). The motif 1 and motifs 2/3 of the VelC *velvet* domain are separated by about 100 aa residues. Unlike in other *Aspergillus* spp., the VelC protein in *A. nidulans* contains one putative PEST domain (ePESTfind, http://emboss.bioinformatics.nl/cgi-bin/emboss/epestfind) which is located between motifs 1 and 2 (392∼406 aa).

### The deletion of velC increases conidiation

**Figure 2:**
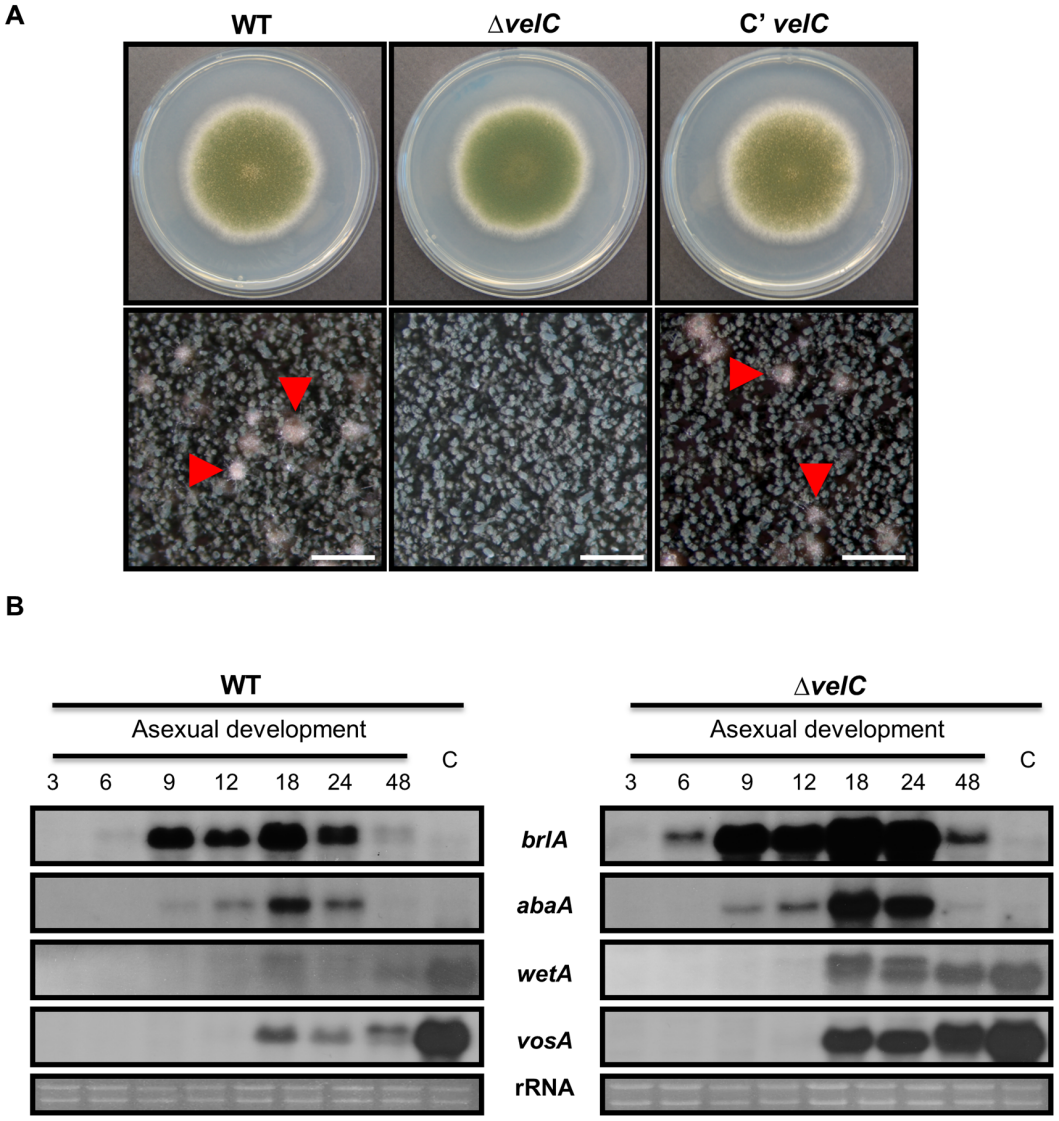
Phenotypes of the Δ*velC* mutant. (A) Colony photographs of WT (FGSC4), Δ*velC* (THS11.1) and complemented (THS25.1) strains point-inoculated on solid MM and grown for 4 days (Top and bottom panels). The bottom panel shows close-up views of the middle of the plates. The sexual fruiting bodies are marked with arrowhead. (bar  =  0.5 mm). (B) Northern blot for *brlA*, *abaA, wetA* and *vosA* mRNAs in WT (FGSC4) and Δ*velC* (THS11.1) strains post asexual developmental induction (Asex). Numbers indicate the time (h) of incubation after induction of asexual development. Equal loading of total RNA was confirmed by ethidium bromide staining of rRNA.

To investigate the role of *velC*, we generated the *velC* deletion (Δ*velC*) mutant and complemented strains, and compared their phenotypes. As shown in Fig. 2A, when point inoculated on solid medium and incubated for 4 days, wild type (WT) and complemented strains started to form sexual fruiting bodies, whereas the Δ*velC* mutant failed to form cleistothecia. We then compared the numbers of conidia and found that the Δ*velC* mutant produced slightly higher number of conidia than WT (data not shown).

To correlate phenotypic changes caused by the absence of *velC* with the molecular events, we examined the mRNA levels of various asexual development-specific genes including *brlA*, *abaA*, *wetA*, and *vosA* in WT and Δ*velC* strains grown under conditions that induce asexual development (Fig. 2B). In WT, accumulation of *brlA* mRNA was detectable at 9 h post developmental induction and reduced after 24 h. In Δ*velC* strain, however, *brlA* mRNA started to accumulate at 6 h, stayed at high levels for 9∼24 h, and remained clearly detectable even at 48 h. In accordance with *brlA* mRNA accumulation patterns, levels of *abaA*, *wetA* and *vosA* mRNA were all higher in the Δ*velC* mutant compared to WT. These results indicate that VelC is necessary for the proper control (down-regulation) of asexual developmental regulatory genes.

### VelC is necessary for proper sexual development

**Figure 3:**
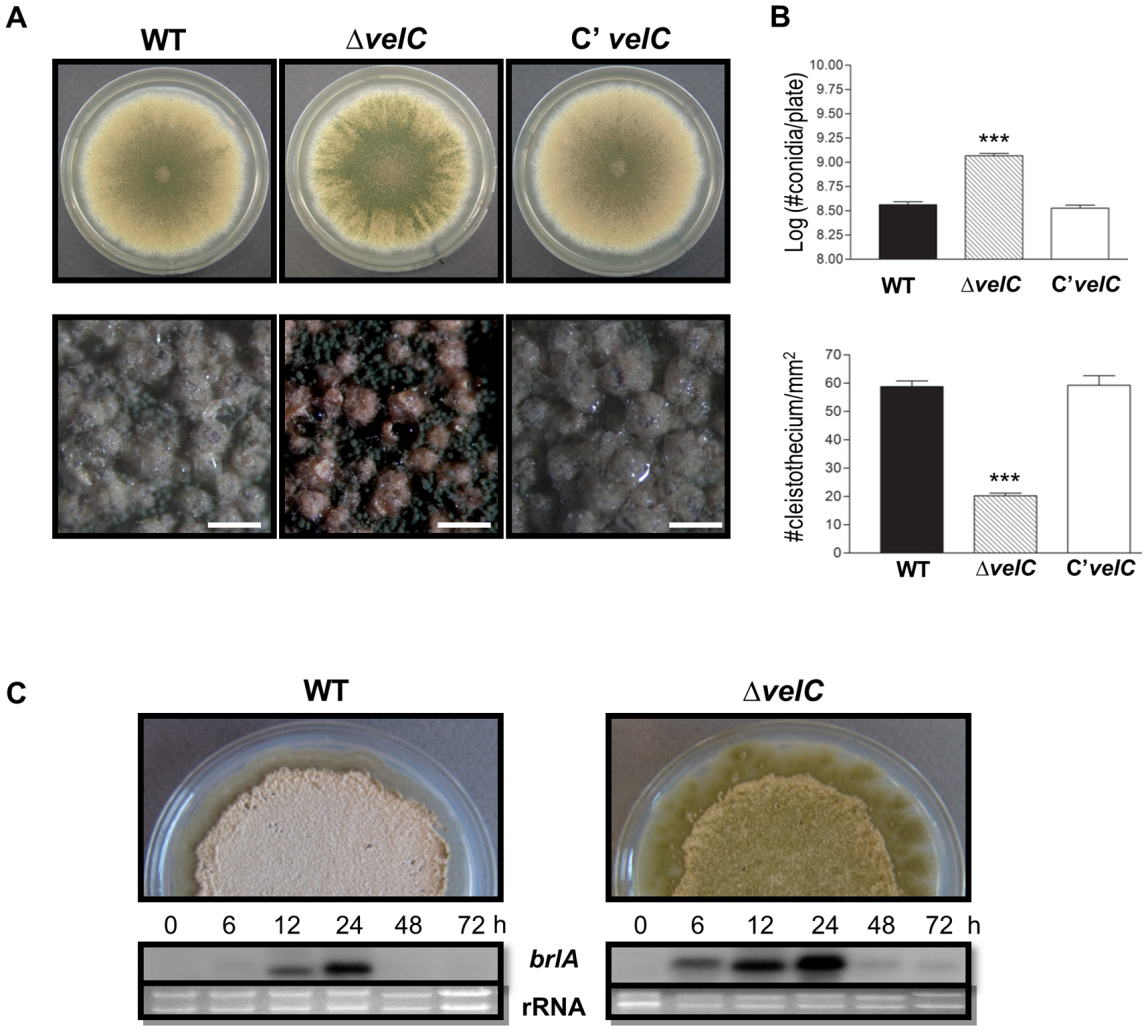
Phenotypes of the Δ*velC* mutant under sexual induction condition. (A) Colony photographs of WT (FGSC4), Δ*velC* (THS11.1) and complemented (THS25.1) strains point-inoculated on solid SM and grown for 7 days (Top and bottom panels). The bottom panel shows close-up views of the middle of the inducing plates. (bar  =  0.5 mm). (B) Quantitative analysis of conidiospores and cleistothecia formation of strains shown in (A) (*** P<0.001). (C) Phenotypes of WT and Δ*velC* strains post sexual developmental induction (Upper panel). Northern blot for *brlA* mRNA level in WT (FGSC4) and Δ*velC* (THS11.1) strains post sexual developmental induction (Sex). Numbers indicate the time (h) of incubation after induction of sexual development. Equal loading of total RNA was confirmed by ethidium bromide staining of rRNA.

As enhanced conidiation can result from the reduced sexual development, we addressed the question whether VelC is associated with activating sexual development. WT, Δ*velC*, and complemented strains were point-inoculated on SM and incubated in the dark under the air-limited conditions for enhancing sexual fruiting for 7 days. As shown Fig. 3B, the Δ*velC* mutant produced significantly reduced number of sexual fruiting bodies compared to WT (P<0.001). Furthermore, the deletion of *velC* resulted in significantly increased conidia production, and high level accumulation of *brlA* mRNA even under the conditions favoring for sexual development (Fig. 3B and C). These results suggest that VelC is required for proper sexual development and balanced progression of asexual and sexual development.

### Overexpression of velC enhances sexual fruiting

**Figure 4:**
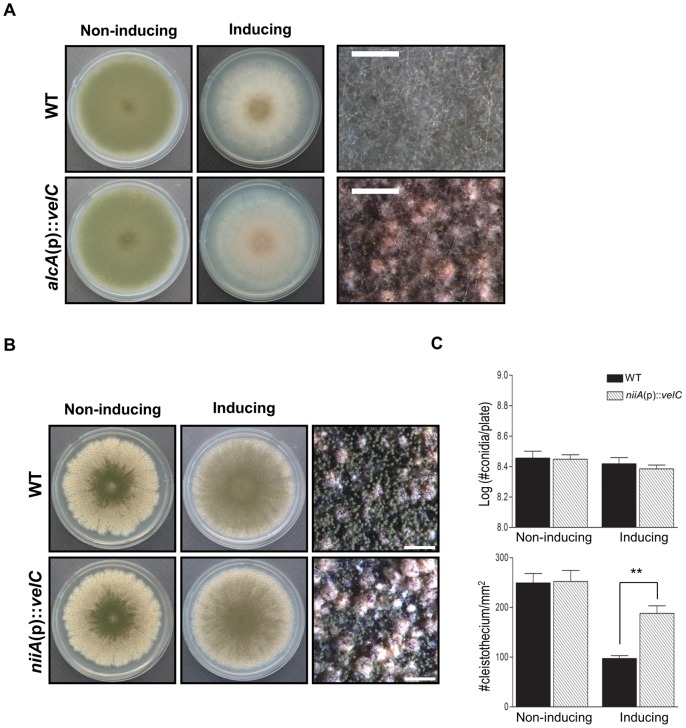
Effects of overexpression of *velC*. (A) WT (FGSC4) and *velC* overexpression (THS 8.1) strains were point inoculated on solid MMG (non-inducing; left panel) or MMT (100 mM threonine, inducing; middle panel) and photographed at day 4. The right panel shows close-up views of the middle of the plates. (bar  =  0.5 mm). (B) WT (FGSC4) and *velC* overexpression (THS23.1) strains were point inoculated on non-inducing (MMG with 0.2% ammonium tartrate) and inducing (MMG with 0.6% sodium nitrate) solid media, and incubated for 4 days. (bar  =  0.5 mm). (C) Effects of overexpression of *velC* in conidiospores and cleistothecia formation. Quantification was done as described in the experimental procedures (** P<0.01).

As described above, the deletion of *velC* resulted in reduced sexual fruiting body production and increased conidiospore production. Two hypotheses regarding the role of VelC can be derived from these results; i) VelC negatively regulates asexual development, which in turn confers sexual development, or ii) VelC positively controls sexual development, which in turn represses conidiation. To address these, we constructed OE*velC* strain (*alcA*(p)::*velC*) by fusing the *velC* ORF with the inducible *alcA* promoter [44]. As shown in Fig 4A, WT strain exhibited a fluffy phenotype and could not produce sexually developing structures on MMT plates. However, OE*velC* strain began to produce Hülle cells (specialized structures supporting sexual fruiting), though did not develop cleistothecia due to the presence of threonine as a sole carbon source, which does not allow sexual development to occur (Fig. 4A). To further examine a potential direct role of VelC in sexual development, the effects of OE*velC* under the *niiA* promoter [31] were examined by growing the individual strains on non-inducing and inducing media. Under non-inducing condition, there were no differences between WT and OE*velC* strains in their cleistothecium and conidiospore production. When point inoculated and cultured under inducing conditions, OE*velC* strain showed two-fold increased production (p<0.01) of sexual fruiting bodies compared to WT, whereas OE*velC* strain produced equivalent amounts of asexual spores compared to WT (Fig. 4B&C). Overall, these results strongly support the idea that the controlled expression of *velC* is necessary for normal fungal development, and that VelC functions as an activator of sexual development.

### VelC is one of the VosA interacting proteins

The *velvet* protein VosA is a multifunctional regulator which plays a complex regulatory role in conidiophore formation and conidia maturation [10], [12], [45]. VosA forms various complexes including homo-dimer or VelB-VosA hetero-dimer which are localized in the nucleus [12], [45]. To better understand the role of VosA, we identified VosA interacting proteins employing yeast-two hybrid assay. The cDNA of VosA (bait) was cloned into pTLexA [42] and the *A. nidulans* cDNA library in pAD-GAL4-2.1 was screened. After carrying out the procedures to remove the false positive candidates, we identified four VosA interacting (Voi) proteins: VoiA (AN10356), VoiC (AN8795), VoiD (AN4252) and VelC (AN2059) (Table 3). VoiA is a hypothetical protein and contains one BTB/POZ domain, which mediates homomeric or heteromeric dimerization [46]. *voiC* encodes the homolog of µ-1 subunit of clathrin-associated adaptor protein (AP) complex 1, which plays a role in protein sorting in the trans-Golgi network (TGN) and endosomes [47]. VoiD is similar to *Histoplasma capsulatum* MS8, which is a mold-specific gene required for normal hyphal formation [48]. VelC is one of *velvet* regulators and contains the *velvet* domain [10].

**Table 3:** VosA interacting protein in *A. nidulans.*

Gene	ORF(locus)	Annotation
*voiA*	AN10356	Uncharacterized protein (BTB/POZ domain)
*voiB*	AN0435	Uncharacterized protein (BTB/POZ domain)
*voiC*	AN8795	AP-1 complex subunit mu-1
*voiD*	AN4252	Uncharacterized protein
*velC*	AN2059	*Velvet* family protein (*velvet* domain)

**Figure 5:**
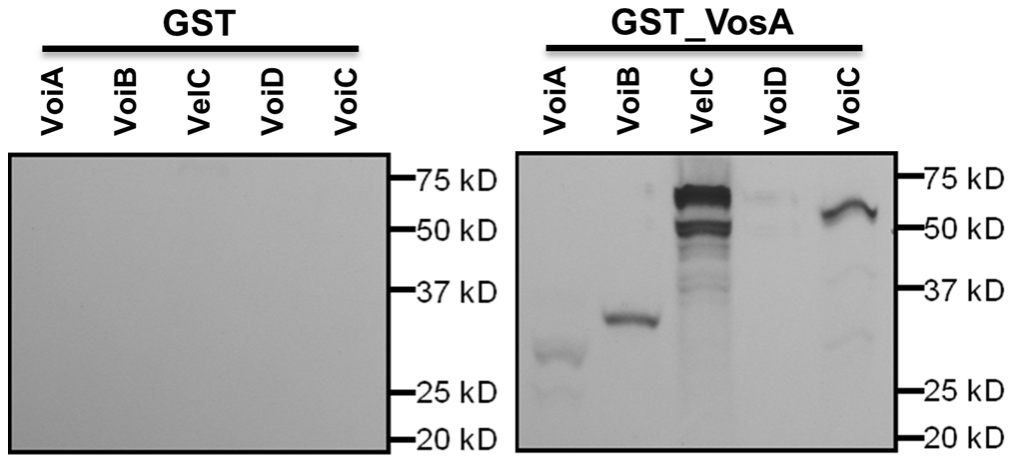
VelC physically interacts with VosA. GST pull down assay for GST or GST-VosA and *in vitro* translated ^35^S-VosA interacting proteins. The *in vitro* translated proteins were divided into two parts (each 20 µl) and mixed with GST alone (right panel) or the GST-VosA protein (left panel). The expected protein size of VoiA, VoiB, VelC, VoiD or VoiC is about 38, 31, 62, 20.9 and 49 kDa, respectively.

To confirm that VosA binds to the Voi and VelC proteins directly *in vitro*, GST-pull down experiments were carried out. In this experiment, we also added a VoiA similar protein, VoiB (AN0435), which contains one BTB/POZ domain in the N-terminal region. The cDNA of the *voi* genes was cloned into pcDNA3, and the Voi proteins were translated *in vitro* and labeled with S^35^. The *vosA* ORF was fused with GST in the pGEX 5X-1 vector, and VosA was expressed in *E. coli* strain BL21 (DE3). Equal amounts of *in vitro* translated proteins were added to glutathione bead-GST-VosA or glutathione bead-GST (control) suspensions and subjected to pull-down. As shown in Fig. 5, S^35^ labeled VoiA∼D and VelC could be co-purified with GST-VosA, indicating that VosA directly binds to these proteins *in vitro*.

### *vosA is* epistatic to *velC*

**Figure 6:**
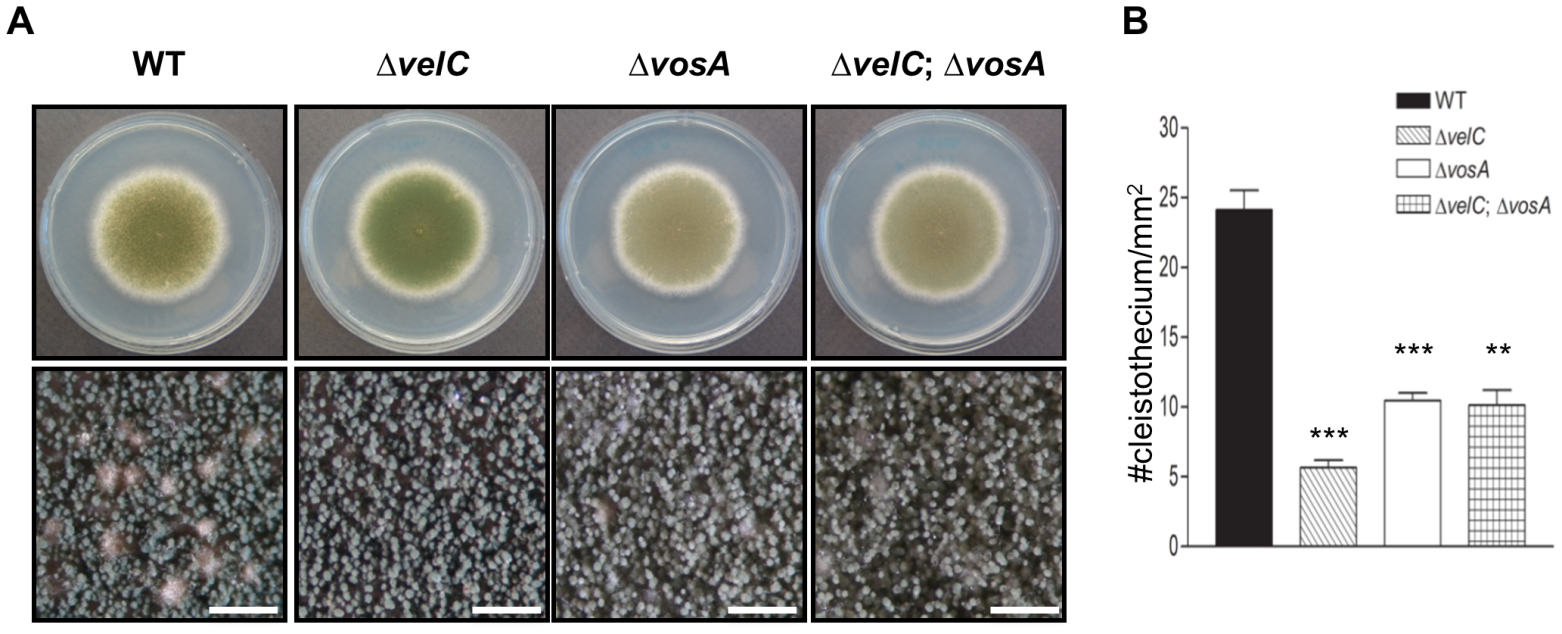
Double mutant analysis. (A) Colony photographs of WT (FGSC4), Δ*velC* (THS11.1), Δ*vosA* (THS15.1) and Δ*velC* Δ*vosA* (THS26.1) strains grown on solid MM for 4 days. (bar  =  0.5 mm). (B) Quantitative analysis of conidiation of strains shown in (A) (** P<0.01; *** P<0.001).

The above data suggest a possible genetic interaction between VosA and VelC. To address this, we generated the *velC* and *vosA* double deletion mutant and compared its phenotypes including asexual and sexual development with the Δ*velC* and Δ*vosA* single mutants. Compared to WT, all three Δ*velC,* Δ*vosA* and Δ*velC* Δ*vosA* mutants exhibited defective sexual fruiting under the air-exposed culture condition, and Δ*vosA* and Δ*velC* Δ*vosA* strains produced light-green conidia typical of the Δ*vosA* mutant (Fig 6A). When point inoculated and cultured for inducing sexual development, the Δ*velC* Δ*vosA* double mutant behaved almost identically to the Δ*vosA* single mutant, slightly enhanced sexual fruiting compared to the Δ*velC* mutant (Fig. 6B). These results suggest that VosA is epistatic to VelC in sexual and asexual development.

## Discussion

The *velvet* family proteins play vital roles in development and secondary metabolism in filamentous fungi [8]. While VeA, VelB and VosA have been characterized in *A. nidulans*, the role of VelC was unclear. In this study, we present the experimental evidence that VelC plays a vital role in controlling asexual and sexual development in *A. nidulans*. The Δ*velC* strain exhibited enhanced production of conidiospores in conjunction with the reduced formation of sexual fruiting bodies (Figs. 2 & 3). Furthermore, examination of mRNA levels of asexual developmental genes suggests that VelC is required for the proper control of asexual specific genes. We then asked whether VelC acts as a repressor of asexual development or an activator of sexual development. First, we examined the phenotypes of the Δ*velC* mutant and found that the Δ*velC* mutant cannot produce conidiophores and induce *brlA* expression in liquid submerged culture (data not shown). Second, OE of *velC* causes elevated production of sexual fruiting bodies. These results indicate that VelC may function as a sexual activator, which indirectly represses conidiation in *A. nidulans.* Unlike OE of *veA* [49], however, OE *velC* strain could not form cleistothecia in liquid cultures. The *velC* gene is expressed specifically during the early phase of sexual development. Taken together, we propose that VelC is a sexual activator which acts during the early phase of sexual development.

The VelC homologues have been characterized in *A. fumigatus*, *A. flavus, Penicillium chrysogenum* and *Fusarium oxysporum* [15], [17], [50], [51]. The Δ*velC* mutant did not show distinct phenotypes in *A. fumigatus* [15] and *A. flavus* [17], suggesting that VelC plays a minor role in asexual development in some *Aspergilli*. In *F. oxysporum*, the Δ*velC* mutant exhibited increased microconidia production and decreased chromatin accessibility [51]. The *velC* homologue in *P. chrysogenum* acts as a repressor of conidiation and activates penicillin biosynthesis [50]. In three fungi, *A. nidulans*, *F. oxysporum* and *P. chrysogenum*, deletion of *velC* caused increased conidia production, suggesting a potential conserved role of VelC in some fungi.

Studies in *F. oxysporum* [51] and *P. chrysogenum* [50] have revealed that VelC physically interacts with other *velvet* regulators. VelC can interact both with VeA and with VelB in *F. oxysporum*. The VeA-VelC complex in *F. oxysporum* plays a negative role in asexual sporulation [51]. In *P. chrysogenum*, VelC also interacts with VelA or VosA and forms two complexes, which localize in the nucleus. Kopke et al. proposed that one multi-subunit *velvet* complex regulates penicillin production and conidiation whereas biological roles of two sub-complexes, VelC-VelA and VelC-VosA, are currently unknown [50]. As found in *P. chrysogenum,* we also identified the *A. nidulans* VelC protein interacts with *A. nidulans* VosA in yeast and *in vitro* (Fig. 5). Most of the phenotypes of the Δ*velC* Δ*vosA* double-deletion mutant, including changes in development, conidial trehalose amount, spore viability and conidial germination, closely resembled those of the Δ*vosA* single deletion mutant, suggesting that *vosA* is epistatic to *velC* in most biological processes.

**Figure 7:**
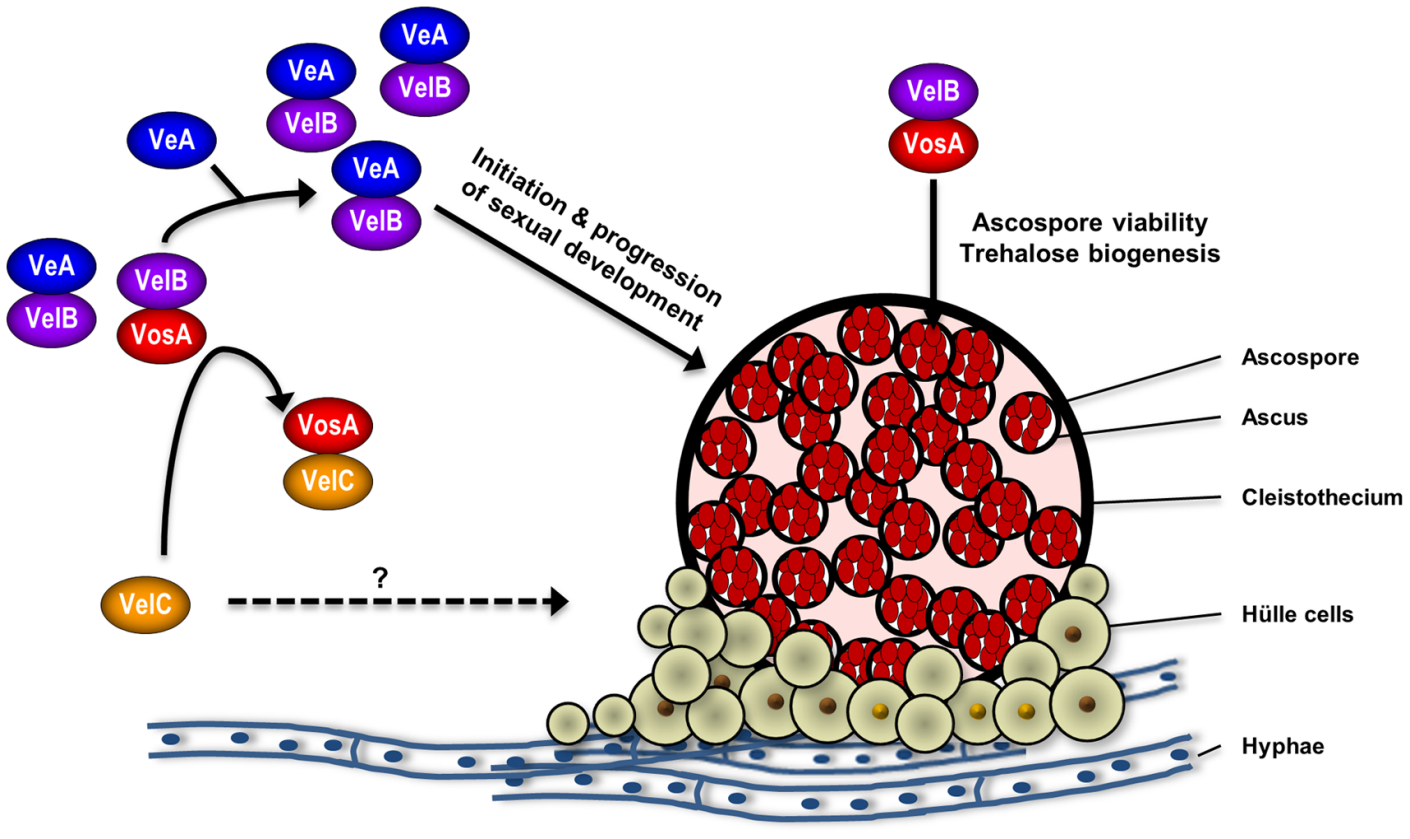
Velvet mediated regulation of *A. nidulans* sexual development. A proposed model for the *velvet* mediated developmental regulation (see Discussion).

Collectively, we propose that the *velvet* proteins or complexes play diverse roles in regulating sexual development in *A. nidulans* (Fig. 7). We can speculate that the dynamic and differential interaction of *velvet*, especially VelB, with its partner may be a key determinant of fungal cellular responses. VelB can form VelB-VelB homo-dimer, VelB-VosA, or VelB-VeA heterodimers [12], [45]. In hyphae, VelB mainly interacts with VeA and forms the VelB-VeA heterodimers which is required for the initiation, progression and completion of sexual development [12]. Some VelB-VosA hetero-complexes may also exist in hyphae. During early phase of sexual development, VelC is produced, which then physically interacts with VosA. Such VelC-VosA interaction leads to increased formation of the VelB-VeA hetero-complex, which then triggers the sexual fruiting process. The VelC protein may also play a potential role in activating sexual development. In ascospores, the VelB protein mainly interacts with VosA [12] and forms the VelB-VosA complex which may play a critical role in regulating trehalose biogenesis and ascospore viability. Further studies revealing the molecular mechanisms of VelC-mediated developmental control will provide novel insights into complex fungal biology.

## Acknowledgments

We thank Dr. Min Ni and Tae Won Kim for technical assistances and Dr. Ellin Doyle for critically reviewing the manuscript.
